# Poster Session II - A271 EVOLUTION OF UC:CD INCIDENCE RATIOS ACROSS EPIDMIOLOGIC STAGES

**DOI:** 10.1093/jcag/gwaf042.271

**Published:** 2026-02-13

**Authors:** J W Windsor, S Coward, L Hracs, J Gorospe, S Okabayashi, G G Kaplan

**Affiliations:** University of Calgary, Calgary, AB, Canada; University of Calgary, Calgary, AB, Canada; University of Calgary, Calgary, AB, Canada; University of Calgary, Calgary, AB, Canada; University of Calgary, Calgary, AB, Canada; University of Calgary, Calgary, AB, Canada

## Abstract

**Background:**

Inflammatory bowel disease (IBD) progresses through distinct epidemiologic stages: Stage 1 (Emergence), low incidence/prevalence; stage 2 (Acceleration in Incidence), rapid increasing incidence; stage 3 (Compounding Prevalence), stabilizing incidence and increasing prevalence. As IBD emerges in a population, ulcerative colitis (UC) incidence typically exceeds Crohn’s disease (CD) incidence, but over time the UC:CD incidence ratio approximates 1.

**Aims:**

To examine changes in UC:CD incidence ratios across epidemiologic stages.

**Methods:**

Stage classifications were previously determined by machine-learning classifier applied to data from 522 population-based studies of 82 countries (1920–2024). Population-weighted mean incidence (excluding zeros) was used to calculate annual, country-specific UC:CD ratios. Ratios were analyzed using a generalized additive model for location, scale, and shape (GAMLSS), accounting for overdispersion and skewness, with pairwise Wilcoxon rank sum tests and Bonferroni correction for between-stage comparisons. Average annual percent changes (AAPC) with 95% confidence intervals (CI) were calculated for each country within each stage. AAPCs were pooled by meta-analysis within stages. Outliers were defined using the Tukey method and are presented separately.

**Results:**

The GAMLSS model, adjusted for country, showed significant effects of epidemiologic stage on UC:CD incidence ratios (*p*<0.001), highlighting a progressive shift toward a 1:1 ratio (Figure 1). Significant differences were found between stages: Stage 1: Median: 3.62 (IQR: 3.9 [1.66–5.56]); Stage 2: Median: 1.8 (IQR: 2 [1.12–3.12]); Stage 3: Median: 1.56 (IQR: 1 [1.08–2.08]); all comparisons: *p*<0.001. Meta-analyses supported these results, decreasing in stages 1 and 2 before stabilizing in stage 3: Stage 1 (13 countries, 1954–2022) AAPC: −4.77 (95%CI: −8.59, −0.95); stage 2 (42 countries, 1952–2022) AAPC: −2.55 (95%CI: −4.04, −1.06); stage 3 (24 countries, 1970–2023) AAPC: −0.12 (95%CI: −0.66, 0.42). Outliers (*n*=81; 7.8%) were primarily observed in Asia and northern latitudes (Table 1) (eg, China, Faroe Islands).

**Conclusions:**

The UC:CD incidence ratio significantly decreases during stage 1 and 2, then stabilizes in stage 3. Recognising stage-specific changes in the UC and CD incidence may clarify the epidemiologic evolution of IBD and guide research into environmental and diagnostic determinants.

A271 Table 1: UC:CD Incidence Ratio Outliers

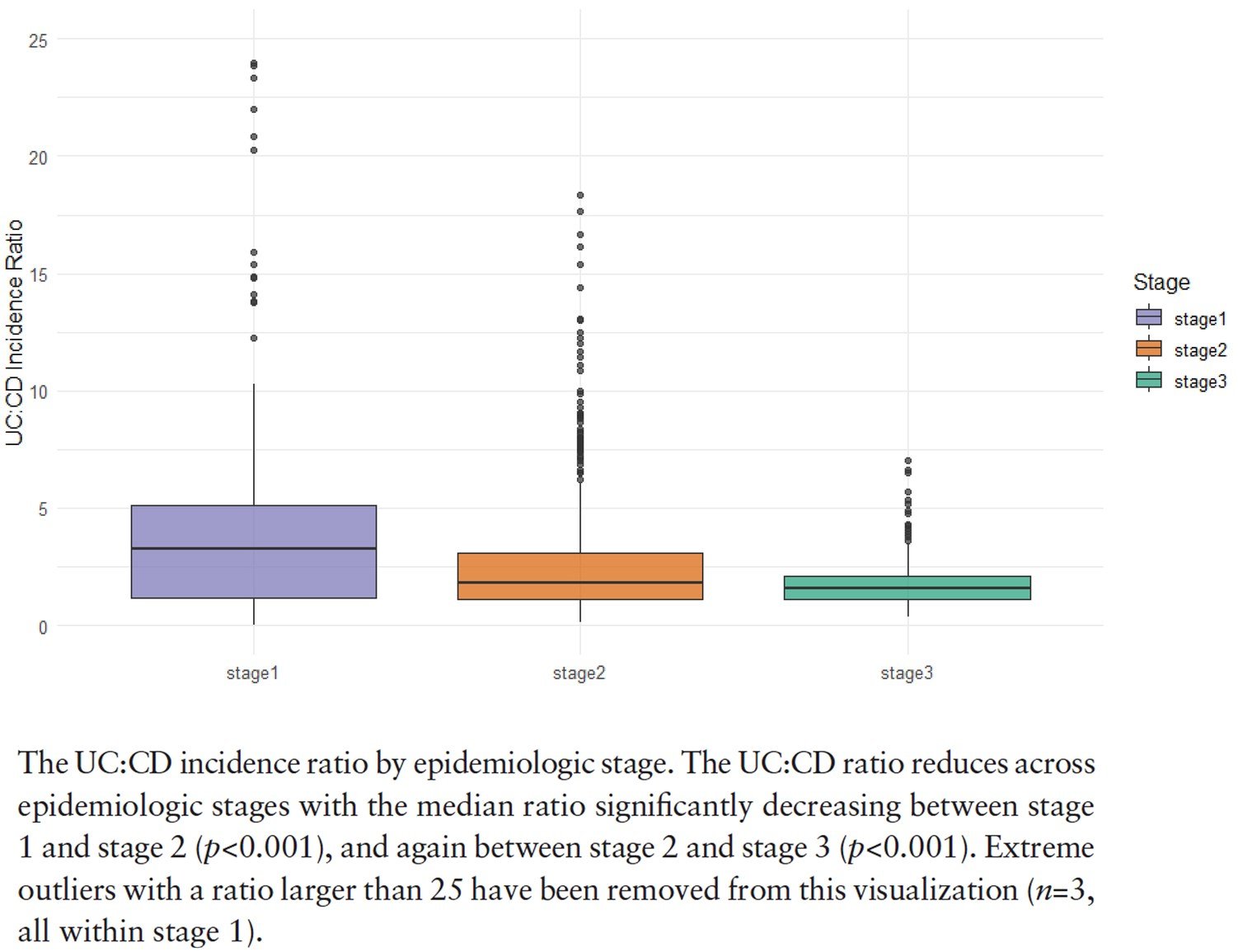

**Funding Agencies:**

CIHRThe Leona M. and Harry B. Helmsley Charitable Trust; International Organization for the study Inflammatory Bowel Disease (IOIBD)

**Table:** 

		**Min**	**Q1**	**Median**	**Q3**	**Max**
**Stage 1 *(n = 17; 10.3%)***	UC Incidence per 100,000	0.025	0.105	0.32	0.403	5.0
	CD Incidence per 100,000	0.002	0.005	0.014	0.025	0.208
	UC:CD Ratio	12.2	14.8	20.3	23.8	83.0
**Stage 2 *(n = 49; 10.96%)***	UC Incidence per 100,000	0.747	3.68	5.35	7.64	21.9
	CD Incidence per 100,000	0.104	0.4	0.6	0.8	2.28
	UC:CD Ratio	6.22	7.62	8.66	11.4	18.3
**Stage 3 *(n = 15; 3.53%)***	UC Incidence per 100,000	10.8	14.2	22.9	43.9	73.7
	CD Incidence per 100,000	2.7	3.51	4.7	7.25	10.8
	UC:CD Ratio	3.63	4.08	4.78	5.49	7.02

